# Dietary supplementation of *Platycodon grandiflorum* polysaccharides mitigates weaning stress in piglets by modulating intestinal microbiota and improving gut health

**DOI:** 10.5713/ab.250877

**Published:** 2026-04-16

**Authors:** Shusen Li, Mingming Cao, Jiaqi Dong, Jiajun Hao, Haoyun Wei, Yantong Guo, Haotian Wang, Xiao Liu, Haoyang Sun

**Affiliations:** 1 College of Animal Science and Technology, Northeast Agricultural University, Harbin, China

**Keywords:** Colon Health, Colon Microorganisms, Growth Performance, Immune Performance, *Platycodon grandiflorum* Polysaccharides, Weaned Piglets

## Abstract

**Objective:**

*Platycodon grandiflorum* polysaccharide (PGP) extracted from *Platycodon grandiflorum*, which has the advantages of anti-inflammatory, antioxidant, anti-tumor, and no side effects on the organism. This study evaluated whether dietary PGP alleviates weaning-induced intestinal dysfunction in piglets.

**Methods:**

A total of 18 newly weaned piglets (6.46±0.31 kg) were allocated to 3 treatments (n = 6/group) for a 28-day feeding trial. The control group received a basal diet, while the L-PGP and H-PGP groups were supplemented with 1 g/kg and 2 g/kg PGP, respectively. On day 28, piglets were electrically stunned and euthanized by exsanguination; spleen, liver, and colon samples were collected to assess inflammatory/antioxidant markers, barrier-related genes, and colonic microbiota.

**Results:**

The supplementation of PGP increased the average daily gain (p<0.05), and reduced the feed-to-gain ratio of weaned piglets. In addition, PGP reduced the expression of splenic IL-1β, IL-2, and IL-4 (p<0.05). This effect was associated with upregulated mRNA levels of the key tight junction proteins Occludin, ZO-1, and Claudin-1 in the colonic mucosa and tissue (p<0.05). Compared with the control group, the mRNA expression levels of PKC, Nrf2 and KEAP1 in the colonic tissue of weaned piglets were increased in the H-PGP (p<0.05). More importantly, H-PGP supplementation increased the relative abundances of *Bacteroidota*, while decreasing the abundance of *Proteobacteria*. Moreover, Spearman correlation analysis revealed that short-chain fatty acid-producing commensal genera (*Faecalibacterium*) were negatively correlated with pro-inflammatory cytokines and signaling genes, but positively correlated with antioxidant genes (p<0.05). Conversely, the relative abundance of *Escherichia–Shigella* was negatively correlated with the mRNA expression of PKC, Nrf2, and Keap1 (p<0.05).

**Conclusion:**

PGP can alleviate the weaning stress in piglets, by modulating the colonic microbiota and enhancing systemic anti-inflammatory capacity and intestinal barrier function.

## INTRODUCTION

In the modern intensive pig farming system, early weaning strategy is widely used to improve sow reproductive performance and production efficiency, thereby enhancing farm economics [1]. However, early weaning of piglets also exposes them to significant health risks. Weaning is one of the most challenging and unavoidable events for piglets [2]. Piglets are exposed to many stresses after weaning, especially in the first week, including transports, diet transition, and environmental changes [3,4]. These changes coincide with the natural decline of maternally derived antibodies and the ongoing maturation of the piglets’ immune system, potentially increasing their susceptibility to environmental challenges [5]. Therefore, during the weaning period, piglets are exposed to multiple stressors. When the intensity of these stressors exceeds the piglets’ adaptive capacity, oxidative stress may occur, which can further lead to intestinal injury and increase the risk of post-weaning syndrome in piglets [6].

At the early stage after weaning, the function of the digestive system of piglets is still in the developmental stage, and intense stress will lead to a decrease in intestinal barrier function, and increased intestinal permeability [7]. The invasion of pathogenic microorganisms can disrupt microbial diversity and the mucosal immune system, and ultimately lead to intestinal inflammation and diarrhea in piglets [8]. Optimal functioning of the digestive system is therefore a key factor in the post-weaning health of piglets. In the interest of food safety and public health, numerous studies have shown that phytobiotics can improve the state of oxidative stress in the organism. For example, *Platycodon grandiflorum* polysaccharide (PGP) supplementation significantly increased the activities of antioxidant enzymes, such as superoxide dismutase (SOD) and glutathione peroxidase (GSH-Px), while decreasing malondialdehyde (MDA) levels in mice subjected to oxidative stress [9]. PGP, extracted from *Platycodon grandiflorum*, is characterized by non-toxicity, wide availability, high biological activity, and easy extraction. From a practical application perspective, PGP can be extracted through a relatively simple and scalable extraction process (hot water extraction followed by alcohol precipitation and purification), which not only enables efficient preparation but also facilitates subsequent research and applications [10]. It has attracted increasing attention in recent years. Studies using mouse models have shown that PGP exhibits significant anti-inflammatory, antioxidant, and anti-apoptotic activities [11,12]. In particular, PGP has been shown to alleviate H_2_O_2_-induced oxidative stress in PC12 cells [13]. Furthermore, PGP could alleviate high-fat diet-induced intestinal damage in mice by affecting gut microbial diversity [14]. However, the current literature does not provide a clear indication of the protective effect of PGP against intestinal damage in piglets caused by weaning stress.

Therefore, the objective of this study was to evaluate the effects of dietary supplementation with low and high doses of PGP on intestinal health in weaned piglets. Additionally, the growth performance, nutrient digestibility, immune factors, and antioxidant regulatory pathways were analyzed. This study will provide evidence in supporting PGP as a dietary strategy to mitigate weaning-induced intestinal injury in piglets.

## MATERIALS AND METHODS

### Experimental animals

A total of 18 healthy weaned Duroc×Landrace×Yorkshire crossbred pigs (average body weight 6.46±0.31 kg) were included in this experiment, with an equal male-to-female ratio (1:1) and no significant individual weight difference (p>0.05). Experimental piglets received vaccinations following the standard immunization protocol of the swine farm, were weaned at 21 days of age, and were introduced to creep feed one week prior to weaning [15].

### Animal experimental design

Throughout the experimental period, piglets were randomly assigned to three treatments, each consisting of six replicates. Piglets were individually housed, with one piglet per pen. Piglets were individually housed in metabolism cages (approximately 1.2 m×0.6 m×0.8 m, length×width×height). Before the experiment, the piglet house was modified according to the requirements of the experimental grouping design, and the water troughs and feed troughs were cleaned, and then the pig house was cleaned and disinfected in all aspects, and the epidemic prevention work was done well. The piglets were vaccinated and weaned at 21 days of age according to the standard immunization procedure of pig farms, and the creep feed was fed one week before weaning. After weaning, piglets were moved to the nursery; temperature was maintained at 25°C–27°C, humidity at 65%–75%, with adequate ventilation to ensure the fresh air in the nursery. Nipple drinkers were installed and checked daily to ensure adequate water supply.

The experiment lasted for 28 days, during which pigs (approximately 21±1 days) were ensured to feed and drink freely every day, and the remaining feed was cleaned up every morning and feed intake was recorded. The experiment was conducted at the A-cheng Experimental Breeding Base of Northeast Agricultural University. Supplement 1 lists the nutrient composition and levels of feeds during animal rearing.

### Sample collection

Following 28 days of the experiment, the piglets (approximately 21±1 days) were electrically stunned and euthanized by exsanguination. Immediately after euthanasia, the thoracic and abdominal cavities were opened, and the heart, spleen, liver, lungs, kidneys, and pancreas were collected and weighed. Within 45 minutes, tissues of the spleen, liver, and distal colon were harvested, rinsed with 0.9% NaCl, blotted dry using filter paper (Xinxing; Hangzhou Special Paper Industry), and stored at −80°C until analysis.

### Measurement of growth performance data

Fasting weights of piglets were measured and recorded at the beginning and end of the trial. Prior to weighing, piglets were fasted for 12 h with free access to water. Feed consumption was meticulously recorded during the feeding period. Average daily gain (ADG), and average daily feed intake (ADFI) were calculated from body weights recorded on days 0 and 28 and daily feed disappearance.

ADG was calculated based on the difference between final and initial body weight divided by the experimental period. ADFI was calculated as total feed consumption divided by the experimental days, and the feed-to-gain ratio (F/G) was calculated as the ratio of feed intake to body weight gain.

### Manure collection and apparent digestibility of nutrients

During the last 4 days of the trial, piglet feces and feed samples were continuously collected (once daily), and nutrient digestibility was determined using the internal indicator method [16]. To ensure sample integrity, fresh uncontaminated feces were collected. During the last three days of the trial, feces were collected in the morning and evening after piglets were fed and impurities were removed from them. The collected feces were added to 10 mL of H_2_SO_4_ (10% v/w) and stored at −20°C in a refrigerator. The fecal samples were removed from the refrigerator before measurement. Prior to measurement, the fecal samples were removed and thawed in a 4°C refrigerator, and the feces from each pig collected over 4 days were mixed well; and 250 g were removed and baked in an oven at 65°C for 72 h. The dried samples were pulverized and sieved through a 40-mesh sieve.

Acid-insoluble ash was measured by incinerating feed and fecal samples to obtain total ash, followed by hydrochloric acid treatment, filtration, secondary incineration, and finally weighing. Determination of nutrients such as moisture, crude protein, crude fiber, neutral detergent fiber, crude fat, calcium content, and phosphorus content in manure and feedstuffs. The formula for determining the final nutrient digestibility of the experiment was as follows [16]:


(1) $$Apparent\;digestibility\;of\;a\;nutrient\;(\%)=\left(1-\frac{b\times c}{a\times d}\right)\times 100$$

where a is the content of a nutrient in the feed; b is the content of a nutrient in the fecal sample; c is the content of acid-insoluble ash in the feed; d is the content of hydrochloric acid-insoluble ash in the fecal sample.

### Organ index measurement

After piglet slaughter, the livers, spleen, hearts, lungs, pancreas and kidneys of the piglets were taken, and the surface of the organs was rinsed with saline and the impurities and water were absorbed with filter paper. The viscera were weighed using a precision electronic scale to obtain the weight of each organ, and the organ index of each organ was calculated; organ index (g/kg) = organ weight/body weight.

### Quantitative reverse transcription polymerase chain reaction

Approximately 0.1 g of colonic mucosa, intestinal tissue (segment), and spleen tissue was accurately weighed, mixed with 1 mL of TRIzol reagent and ceramic grinding beads, and then transferred into a homogenization tube for mechanical disruption. Grinding was performed in a mill with a parameter of 4,000 r/min for 10 min. After resting for 20 minutes, the samples were centrifuged in a centrifuge at 12,000 r/min for 15 minutes and the supernatant was transferred to an RNAase-free tube. One-quarter volume of chloroform was added to the tube and mix thoroughly. The well-mixed samples were placed in a centrifuge and the parameters were set to 12,000 r/min and centrifuged at 4°C for 15 min. The supernatant was taken with a new RNAase-free tube and isopropyl alcohol solution in the same volume was added as the supernatant and mixed thoroughly. The samples were allowed to stand for 10 min at 4°C and then centrifuged for 15 min in a centrifuge with the same settings as above. The supernatant was then removed and 1 mL of the configuration reagent (anhydrous ethanol: DEPC = 3:1) was added to the test tube to completely dissolve the precipitate. Dissolved samples were placed in a centrifuge and centrifuged at 7,500 r/min for 5 minutes at 4°C. The supernatant was discarded and 40 μL of DEPC was added to dissolve the samples. The purity and quality of RNA in the prepared samples were measured by ultramicro spectrophotometer (Implen). As it was required that the concentration of RNA in the prepared samples met the preset standard, with the A260/A280 ratio constrained between 1.8 and 2.0. RNA from the samples was reverse transcribed into cDNA using a reverse transcription reagent. The polymerase chain reaction (PCR) amplification of cDNA was performed using the ABI 7500 Polymerase Chain System according to the reagents provided by the reagent vendor (Takara). The relative expression of target genes was calculated using the 2^−ΔΔCt^ method with β-actin as reference [17]. The primers used were synthesized by Sangon Biotechnology based on GeneBank target gene sequences. Primer information is shown in (Supplement 2).

### Microbiota analysis

The DNA samples from the colon contents were extracted using the E.Z.N.A. Stool DNA Kit (Omega Bio-tek). Then, DNA quality was detected using agarose gel electrophoresis. Subsequently, the sample DNA was quantified using a UV spectrophotometer (NanoDrop 2000; Thermo Fisher Scientific).

The sequences that can respond to the specificity of the bacterial colony were used as targets, and the corresponding primers were designed for PCR amplification. The PCR reaction conditions are shown in Supplement 3, and the reaction system is shown in Supplement 4.

The PCR products were obtained by purification with AMPure XT beads. Paired-end sequencing (2×250 bp) was performed on the Illumina NovaSeq 6000 platform. Afterwards, the raw data were filtered and merged using fqtrim (ver. 0.94). Taxonomic assignment was performed against the SILVA v138 database. The UPARSE (ver. 7.1) was used to cluster sequence fragments into operational taxonomic units (OTUs). Alpha-and beta-diversity analyses were conducted with QIIME2 2023.9. The relative abundance of the flora was visualized using R software (ver. 3.3.3).

### Data analysis

Raw data were collated using Microsoft Excel. The data were then analyzed for significance using SPSS one-way analysis of variance (ANOVA) (IBM-SPSS). All results are expressed as mean±standard error of the mean (SEM). Data were analyzed by one-way ANOVA with treatment as the fixed effect. When a significant treatment effect was detected, means were compared using Tukey’s multiple comparison test. Data were tested for normality and homogeneity of variance prior to analysis. No data points were excluded unless attributable to clear analytical or procedural errors. Statistical significance was declared at p<0.05, and 0.05≤p<0.10 was considered a trend.

## RESULTS

### Effect of *Platycodon grandiflorum* polysaccharide on the growth performance of weaned piglets

The results of the experiment are shown in Table 1, compared to the CON group, dietary H-PGP increased the ADG and final body weight (p<0.05). The L-PGP group exhibited a tendency to increase the ADG of weaned piglets, while the ADFI showed a slight decrease (p<0.10). More importantly, the F/G was decreased in the H-PGP group (p<0.05).

### Effect of adding *Platycodon grandiflorum* polysaccharide to the diet on organ weight relative to live body weight

Dietary supplementation with PGP increased pancreas weight and spleen weight relative to live body weight in weaned piglets; however, the differences were not statistically significant (data not shown). No differences were observed in the remaining parameters.

### Effect of dietary supplementation with *Platycodon grandiflorum* polysaccharide on nutrient digestibility

The results are shown in (Table 2). H-PGP group significantly enhanced nutrient digestibility of crude fiber in the diets (p<0.05). Compared to the CON group, L-PGP and H-PGP significantly (p<0.05) increased the apparent total tract digestibility of NDF, CP, Ca and P in weaned piglets, but there is no difference between the PGP treatments. In addition, experimental L-PGP group increased (p<0.05) the apparent total tract digestibility of ether extract.

### Effect of dietary supplementation of *Platycodon grandiflorum* polysaccharide on mRNA expression related to spleen inflammation in weaned piglets

**Figure 1:**
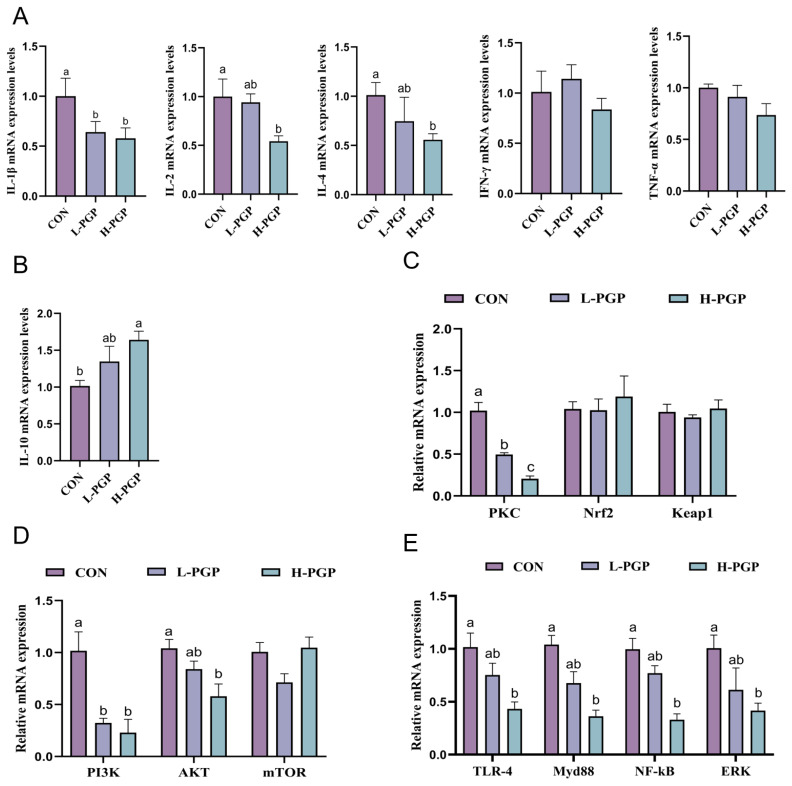
PGP on the expression of spleen inflammatory factors and Genes involved in inflammatory responses in piglets. (A, B) mRNA expression of inflammatory cytokines in the spleen; (C) PKC/Nrf2/Keap1 signaling pathway; (D) PI3K/Akt/mTOR pathway-related genes; (E) TLR4/MyD88/NF-κB signaling pathway genes. Data are expressed as mean±SEM (n = 6). CON: control group; L-PGP: low-dose *Platycodon grandiflorum* polysaccharide group; H-PGP: high-dose *Platycodon grandiflorum* polysaccharide group. ^a,b^ Different lowercase letters represent significant differences at p<0.05. IL-1β, interleukin 1β; IL-2, interleukin 2; IL-4, interleukin 4; IFN-γ, type II interferon; TNF-α, tumor necrosis factor-alpha; IL-10, interleukin 10; PKC, protein kinase C; Nrf2, nuclear factor E2-related factor 2; Keap1, Kelch ECH-related protein 1; PI3K, phosphatidylinositol3-kinase; AKT, serine-threonine kinase; mTOR, mammalian target of rapamycin; TLR-4, toll-like receptor 4; Myd88, myeloid differentiation factor 88; NF-κB, nuclear factor κb; ERK, extracellular regulated protein kinases.

As shown in Figures 1A, 1B, H-PGP decreased the expression of IL-1β, IL-2, and IL-4 and increased the expression of IL-10 (p<0.05). Subsequently, we analyzed variation in the mRNA expression levels of pathways associated with inflammatory response. The mRNA expression of PKC and PI3K was reduced in the PGP groups (p<0.05), while the mRNA expression of Akt, TLR4, MyD88, NF-κB, and ERK was decreased in the H-PGP group (Figures 1C–1E; p<0.05). Unfortunately, the remaining indicators in the L-PGP group were not different from the control group.

### Effect of dietary *Platycodon grandiflorum* polysaccharide on the expression of mRNA related to colonic mucosal inflammation

**Figure 2:**
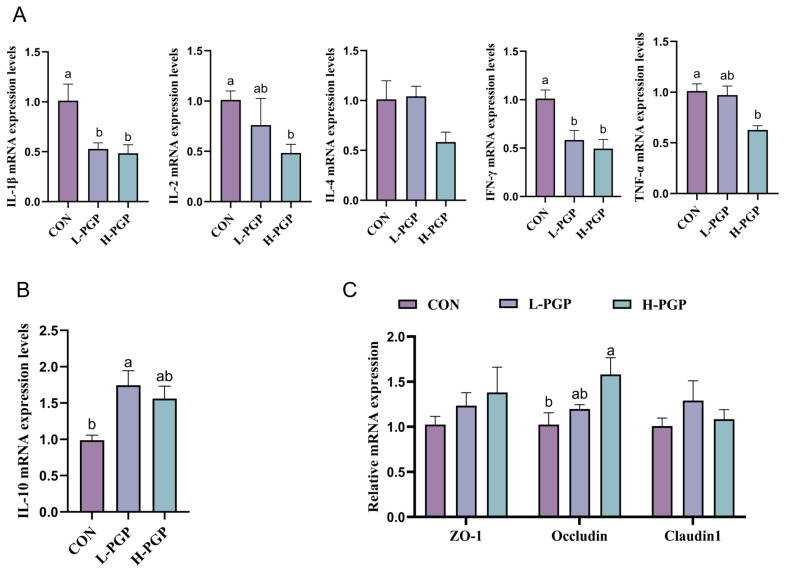
Effect of PGP on the expression of inflammatory factors and mRNA of genes related to intestinal barrier function in the colonic mucosa of piglets ZO-1. (A, B) Pro- and anti-inflammatory cytokine mRNA expression in the colonic mucosa (C) mRNA expression of intestinal barrier–related genes (tight junction proteins). Data are expressed as mean±SEM (n = 6). CON: control group; L-PGP: low-dose *Platycodon grandiflorum* polysaccharide group; H-PGP: high-dose *Platycodon grandiflorum* polysaccharide group. ^a,b^ Different lowercase letters represent significant differences at p<0.05. PGP, *Platycodon grandiflorum* polysaccharide; SEM, standard error of the mean.

**Figure 3:**
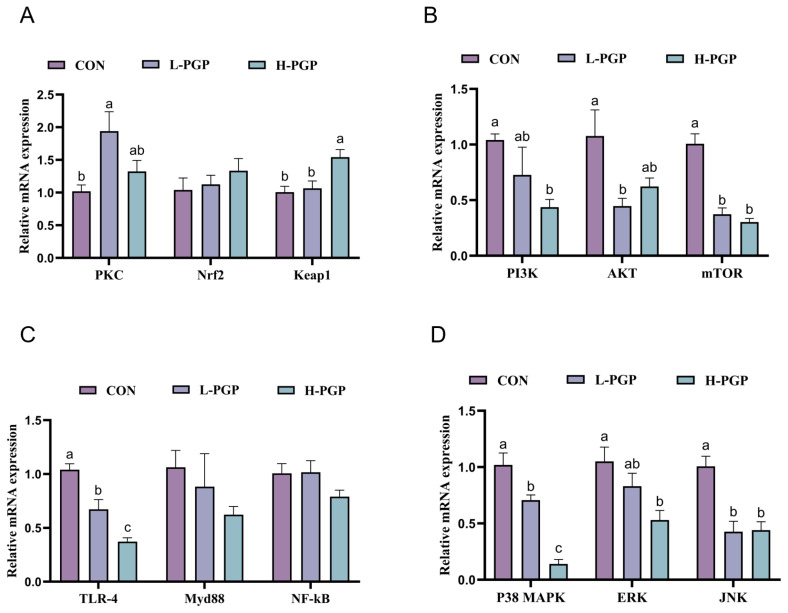
Effects of PGP supplementation on the mRNA expression of genes related to inflammatory signaling pathways in the colonic mucosa of piglets. (A) Oxidative stress–related genes (PKC, Nrf2, and Keap1). (B) PI3K/Akt/mTOR signaling pathway–related genes. (C) TLR4/Myd88/NF-κB signaling pathway–related genes. (D) MAPK signaling pathway–related genes (p38 MAPK, ERK, and JNK). Data are expressed as mean±SEM (n = 6). PGP: L-PGP group+H-PGP group. CON: control group; L-PGP: low-dose *Platycodon grandiflorum* polysaccharide group; H-PGP: high-dose *Platycodon grandiflorum* polysaccharide group. ^a–c^ Different lowercase letters represent significant differences at p<0.05. PKC, protein kinase C; Nrf2, nuclear factor E2-related factor 2; Keap1, Kelch ECH-related protein 1; PI3K, phosphatidylinositol3-kinase; AKT, serine-threonine kinase; mTOR, mammalian target of rapamycin; TLR-4, toll-like receptor 4; Myd88, myeloid differentiation factor 88; NF-κB, nuclear factor κb; ERK, extracellular regulated protein kinases; SEM, standard error of the mean.

As shown in Figures 2A, 2B, PGP decreased (p<0.05) the mRNA expression of IL-1β and IFN-γ. Dietary H-PGP diminished (p<0.05) the mRNA expression of IL-2 and TNF-α. Dietary L-PGP raised the mRNA expression of IL-10 (p<0.05). The results evidenced that PGP could alleviate the inflammation of piglets due to weaning stress. In an effort to assess the protective effect of PGP on the intestinal health of weaning stress in piglets. This study examined the barrier function and the expression of mRNAs mediating pathways related to the inflammatory response (Figure 2C). The PGP decreased the expression of mRNAs for mTOR, TLR4, p38MAPK, and JNK (Figures 3A–3D) whereas L-PGP elevated the mRNA expression of PKC and reduced the mRNA expression of Akt (Figures 3A, 3B; p<0.05). Additionally, the H-PGP group elevated the mRNA expression of Occludin and Keap1, and reduced the mRNA expression of ERK (Figures 2C, 3A, 3B; p<0.05).

### Effect of dietary *Platycodon grandiflorum* polysaccharide on mRNA expression related to colonic inflammation in weaned piglets

**Figure 4:**
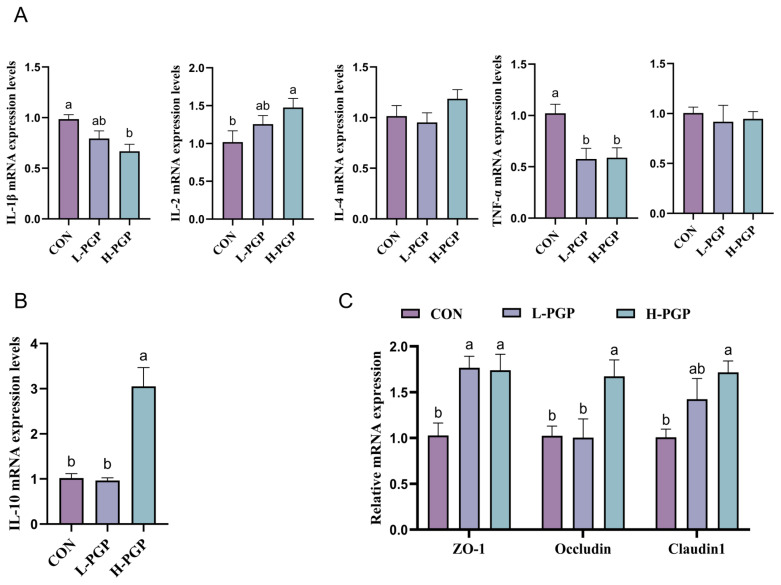
Effect of PGP on the expression of colonic inflammatory factors (A, B) and mRNA (C) of genes related to intestinal barrier function in piglets. Data are expressed as mean±SEM (n = 6). PGP: L-PGP group+H-PGP group. CON: control group; L-PGP: low-dose *Platycodon grandiflorum* polysaccharide group; H-PGP: high-dose *Platycodon grandiflorum* polysaccharide group. ^a,b^ Different lowercase letters represent significant differences (p<0.05). SEM, standard error of the mean.

The expression of mRNAs related to colonic inflammatory factors and intestinal barrier function in piglets was shown in (Figures 4A–4C). Compared with the CON group, the mRNA expression differences of IL-1β, IL-2, IL-10, Occludin, and Claudin-1 in the H-PGP group were different (p<0.05). The PGP group increased the mRNA expression of ZO-1 but decreased the mRNA expression of TNF-α (p<0.05).

**Figure 5:**
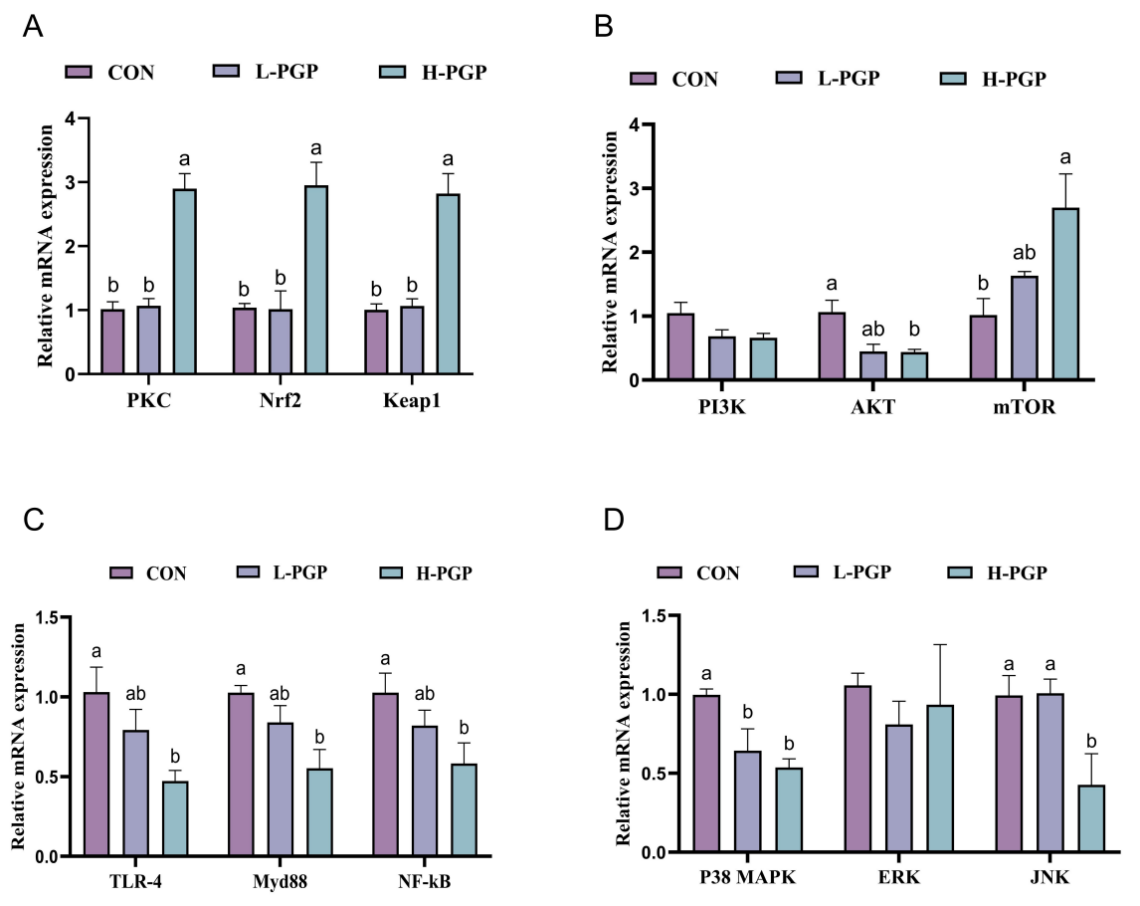
Effects of PGP supplementation on the mRNA expression of genes related to inflammatory signaling pathways in the colonic of piglets. (A) mRNA levels of Nrf2/Keap1 pathway-related genes (PKC, Nrf2, Keap1); (B) mRNA levels of PI3K/AKT/mTOR pathway-related genes (PI3K, AKT, mTOR); (C) mRNA levels of TLR4/NF-κB pathway-related genes (TLR4, MyD88, NF-κB); (D) mRNA levels of MAPK pathway-related genes (P38 MAPK, ERK, JNK). Data are expressed as mean±SEM (n = 6). PGP: L-PGP group+H-PGP group. CON: control group; L-PGP: low-dose *Platycodon grandiflorum* polysaccharide group; H-PGP: high-dose *Platycodon grandiflorum* polysaccharide group. ^a,b^ Different lowercase letters represent significant differences at p<0.05. PKC, protein kinase C; Nrf2, nuclear factor E2-related factor 2; Keap1, Kelch ECH-related protein 1; PI3K, phosphatidylinositol3-kinase; AKT, serine-threonine kinase; mTOR, mammalian target of rapamycin; TLR-4, toll-like receptor 4; Myd88, myeloid differentiation factor 88; NF-κB, nuclear factor κb; ERK, extracellular regulated protein kinases; SEM, standard error of the mean.

To investigate the alleviation of weaning stress-induced colonic inflammation by PGP, the expression of relevant mRNAs mediating the inflammatory pathway was examined in this study. Dietary H-PGP boosted the mRNA expression of PKC, Nrf2, Keap1, and mTOR (Figures 5A, 5B) and decreased the mRNA expression of TLR4, MyD88, and NF-κB (Figures 5B, 5C; p<0.05). Additionally, the PGP Akt group reduced the mRNA expression of p38MAPK (Figure 5D; p< 0.05). Unfortunately, the rest of the indicators in the L-PGP group were not different compared with the control group.

### Effect of *Platycodon grandiflorum* polysaccharide on the microbiota of colonic contents in piglets

**Figure 6:**
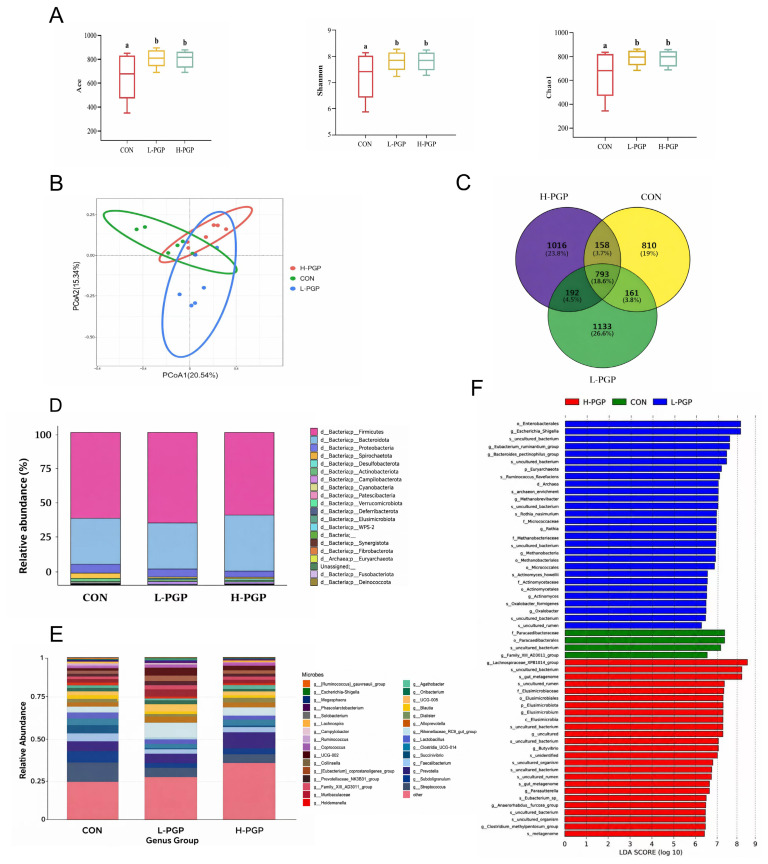
Effect of PGP on microbiota composition of colonic contents. Comparison of ACE index, Chao1, Shannon index (A) colon content beta diversity (B) and Venn diagrams of shared and specific OTUs (C) observed in the microbiota of the colon contents of the control and PGP groups. Relative abundance of fecal microbial phylum (D), genus (E). and analysis of differences at the genus level of colon contents (F) (n = 6 per group). CON: control group; L-PGP: low-dose *Platycodon grandiflorum* polysaccharide group; H-PGP: high-dose *Platycodon grandiflorum* polysaccharide group. ^a,b^ Different lowercase letters represent significant differences at p<0.05. PGP, *Platycodon grandiflorum* polysaccharide; OTUs, operational taxonomic units.

**Figure 7:**
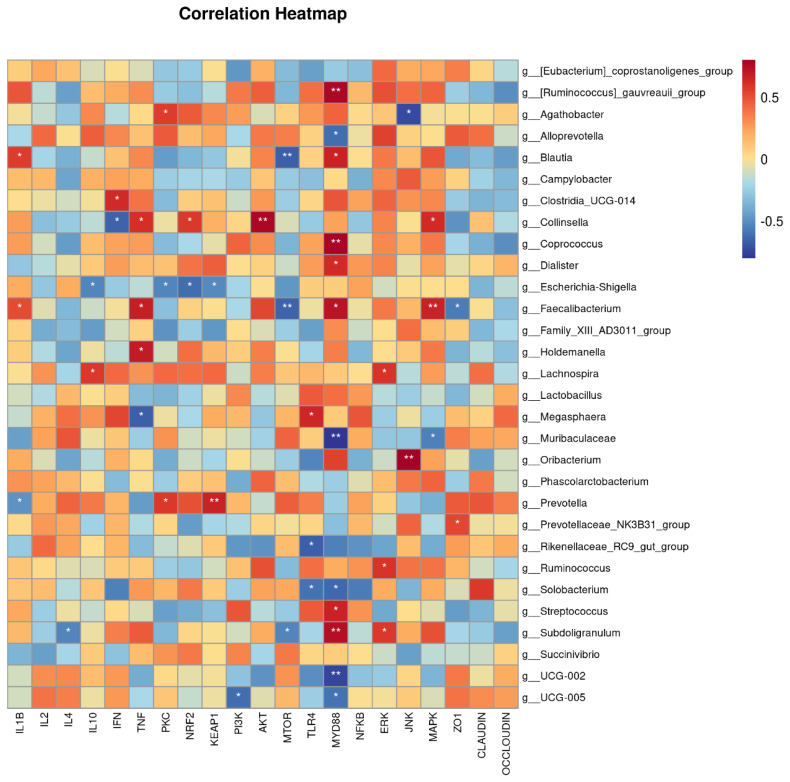
Spearman’s correlation between the concentrations of factors associated with inflammation and oxidative stress and the relative abundance of microbial genera in the control and PGP groups. r values are shown in different colors, with red and blue indicating positive and negative correlations, respectively: * p<0.05, ** p<0.01. PGP, *Platycodon grandiflorum* polysaccharide.

Explore the potential mechanism by which PGP plays an optimal ameliorative role in alleviating colonic inflammation induced by weaning stress in piglets. In this study, 16S rRNA gene sequencing of colonic contents was conducted to analyze how PGP affects the microbial composition of colonic contents. The results are shown in (Figure 6). The β-diversity and OTU Venn diagrams were illustrated between the three groups (Figures 6B, 6C). The relative abundance of Bacteroidota increased, while Proteobacteria decreased in PGP treated groups (Figure 6D; p<0.05). Immediately after this, we analyzed the bacterial genus level in the colonic contents and performed an ANOVA (Figures 6E, 6F). We observed differences in the abundance of 25 bacterial genera in the L-PGP and 31 bacterial genera in the H-PGP. We further analyzed the relationship between colon content flora and factors associated with regulating oxidative stress and inflammation using Spearman correlation analysis. The results are shown in Figure 7.

## DISCUSSION

Antibiotics have been extensively used in animal husbandry over the past few decades to ensure healthy animal growth. This leads to the development of drug resistance in an increasing number of pathogens to the extent that it poses a huge challenge to human health globally [18]. Natural plant extracts with fewer residues, no drug resistance and minimal side effects are gradually entering the livestock industry [19]. Previous studies have reviewed the antioxidant and anti-inflammatory activities of PGP *in vitro* experiments, but little has been reported on the protective mechanisms against stress in piglets due to early weaning. Therefore, we chose early weaned piglets as an animal model to evaluate the protective mechanism of PGP. We found that PGP could improve the immunocompetence of immune organs, colon antioxidant capacity, composition of intestinal contents and microecological composition in early weaned piglets. This provides new insights into finding alternatives to antibiotics.

The ADG and ADFI are macro-indicators that reflect the growth condition and health status of pigs, and these indicators directly affect the economic benefits of pig farms. Weaning stress can lead to loss of appetite, decreased feed intake, poor absorption and digestion of piglets, resulting in growth and development being affected. Previous studies indicate that natural plant polysaccharides can alter the growth performance of piglets, chickens and other animal models [20,21]. Similar to previous results, the addition of both low and high doses of PGP to the diet improved the growth performance of piglets, as evidenced by increases in ADG and a reduction in F/G. Additionally, the organ index of an animal is an indicator of the development and health condition of the organism. The spleen is a major immune organ rich in various immune cell populations. In the present study, both low and high doses of PGP tended to increase spleen weight relative to live body weight, although the differences were not statistically significant. This change may reflect a modulation of immune status rather than an adverse response. Similar observations have been reported in studies evaluating immune organ development in mice [22]. Increased production of inflammatory cytokines as well as decreased production of anti-inflammatory cytokines in the spleen predicts the onset of inflammation in the body [23].

The TNF-α, IL-1β, IL-6, and IFN-γ are the most typical pro-inflammatory cytokines, whereas IL-2 and IL-4 are immune cell secreted factors, and IL-10 is a key anti-inflammatory factor [24]. It has been documented that PGP alleviates RSV-induced respiratory inflammation by reducing the levels of IL-1β, IL-4, IL-6, TNF-α, and IFN-γ [25]. In this study, PGP decreased the mRNA expression of splenic IL-1β, IL-2, and IL-4, while IL-10 was increased, which is considered a self-protective mechanism of the organism [26]. The TLR4 is reported to be an important signaling receptor involved in the regulation of inflammatory responses through MyD88- and NF-κB–related pathways [27]. We observed downregulation of Akt, MyD88, TLR4, and NF-κB mRNA. These results suggest that PGP supplementation was associated with reduced expression of pro-inflammatory cytokines and TLR4-related signaling molecules in the spleen. The downregulation of TLR4 expression may be related to decreased activity of the MyD88/NF-κB inflammatory pathway and PI3K/Akt signaling pathway. After weaning of piglets due to dietary changes, it is likely to cause the destruction of intestinal barrier function, increasing intestinal permeability. This provides the conditions for toxins to cross the mucosal layer and penetrate the organism, thereby causing intestinal inflammation and disrupting microbial balance [28]. Intestinal barrier function is strongly associated with tight junction proteins such as Occludin, Claudin-1, ZO-1, and junctional adhesion molecules [29].

Intestinal barrier dysfunction is often linked to intestinal inflammation. We found that high doses of PGP significantly reduced the mRNA expression of IL-1β and IL-2 in the colon and increased the expression of IL-10. This was confirmed in a mouse liver study [30]. The changes in IL-2 and IL-4 observed in this study may be associated with modulation of immune cell activity during weaning stress. In conclusion, PGP was associated with alleviation of weaning stress–induced colonic inflammation. *Platycodon grandiflorum* extract has been reported to limit oxidative stress and alleviate skin inflammation by activating the Nrf2 pathway and inhibiting NF-κB [31]. We observed that high-dose PGP increased the expression of PKC, Nrf2, and KEAP1. The PKC phosphorylates Nrf2, dissociates Nrf2 from KEAP1, promotes the nuclear translocation of Nrf2, promotes the transcription of antioxidant genes, and alleviates oxidative stress [32].

The PI3K/Akt signaling pathway is one of the conditions for the activation of Nrf2 [33]. This may be due to the fact that PI3K signaling molecules are regulated by multiple pathways whereas PGP decreased the expression of PI3K and Akt in this study. Notably, when the body is subjected to exogenous pathogens or inflammatory stimuli TLR4 activates MyD88-dependent and MAPK signaling pathways to mediate the inflammatory response. In the colon, high-dose PGP significantly reduced the expression of TLR4, MyD88, p38MAPK, ERK, and JNK, and these results were consistent with Zhou et al [34].

In addition, PGP is primarily composed of arabinose, galactose, and glucose. The specific monosaccharide composition and their branched structural configuration are closely associated with their biological functions. In particular, the presence of acidic functional groups (−COOH) enhances free radical scavenging capacity through electron-donating effects, thereby contributing to improved antioxidant activity [35]. Collectively, these key chemical constituents and structural characteristics likely act synergistically to mediate the physiological responses observed in the present study.

In the early stages of weaning when microbial profiles have not yet stabilized, regulating the microbiota in response to this “window of opportunity” is essential to alleviate weaning stress in piglets [36]. PGP treatment significantly increased the abundance of colonic microorganisms and altered colonic microbial diversity and community structure. The microbiologically dominant groups in the PGP-treated colon contents in this study were Firmicutes, Bacteroidota, Proteobacteria, Actinobacteriota, and Campylobacterota, with a relative abundance of more than 95%. The sum of the abundances of major phyla was above 95%, and the relative abundance of Bacteroidota increased while that of Proteobacteria decreased in the PGP group compared to the control group. Bacteroidota bacteria in the pig colon have been reported to use the colon contents as a carbon source and ferment to produce SCFAs, which are sufficient to provide 5%–20% of the body’s energy supply [37]. We found that PGP increased Bacteroidota, a phylum enriched for polysaccharide-utilization capabilities, consistent with improved fiber digestibility (Figure 6D). Studies have shown that the phylum Proteobacteria reflects microecological dysbiosis in the intestine [38], and that it is also associated with the pathogenesis of intestinal inflammation. The above results showed that PGP was able to improve the composition of microorganisms in the colon of weaned piglets, prevent the proliferation of harmful bacteria, and promote the colonization of probiotics, which further indicated that it could promote the intestinal nutrient absorption of weaned piglets and alleviate the damage brought by weaning stress to the intestinal tract of piglets. The correlation heatmap revealed significant associations between intestinal microbiota and host inflammatory signaling pathways. Potentially beneficial genera, such as Lactobacillus, were negatively correlated with TLR4, MyD88, and NF-κB expression, consistent with reports that Lactobacillus can suppress TLR4/NF-κB–mediated inflammatory responses [39]. In addition, the relative abundance of *Escherichia–Shigella* was negatively correlated with the mRNA expression of PKC, Nrf2, and Keap1. This finding is consistent with previous research showing that the relative abundance of Escherichia–Shigella was negatively correlated with the expression of genes in the Keap1/Nrf2/HO-1 signaling pathway [40].

## CONCLUSION

In summary, dietary addition of PGP alleviated the inflammatory colonic response induced by early weaning of piglets. PGP reduced the levels of inflammatory factors in the spleen, colon, and colonic mucosa. In addition, it improved intestinal barrier function and microbial composition. Dietary PGP mitigated weaning-associated colonic inflammation, and this protective effect is presumably mediated by inhibiting the TLR4-MyD88-NF-κB and MAPK signaling cascades while activating colonic antioxidant pathways, such as the Nrf2 pathway. Thus, it was demonstrated that the natural plant extract PGP can be used as an effective dietary supplement to relieve the negative effects of weaning stress on piglet growth and development.

## Figures and Tables

**Table 1:** Effect of dietary supplementation of PGP on growth performance of weaned piglets

Item	CON	L-PGP	H-PGP
Initial BW (kg)	6.43±0.09	6.51±0.02	6.45±0.07
Final BW (kg)	15.80±0.38^b^	16.13±0.25^ab^	16.73±1.01^a^
ADG (kg/d)	0.33±0.02^b^	0.35±0.01^ab^	0.38±0.03^a^
ADFI (kg/d)	0.64±0.03	0.67±0.13	0.66±0.07
F/G	1.94±0.11^b^	1.90±0.06^ab^	1.74±0.09^a^

CON: control group; L-PGP: low-dose *Platycodon grandiflorum* polysaccharide group; H-PGP: high-dose *Platycodon grandiflorum* polysaccharide group.

a,b Using p<0.05 as the criterion of significant difference, values with different lowercase letters superscripts mean significant difference.

BW, body weight; ADG, average daily gain; ADFI, average daily feed intake; F/G, feed-to-gain ratio.

**Table 2:** Effect of the addition of PGP in the diet on the apparent digestibility of weaning piglets

Item	CON	L-PGP	H-PGP
DM (%)	83.4±6.3^b^	84.1±5.2^ab^	85.1±3.8^a^
CF (%)	37.2 ±6.1^b^	39.7±5.3^a^	41.4±4.4^a^
NDF (%)	39.3±6.2^b^	40.1±1.7^a^	41.8±1.9^a^
CP (%)	86.6±1.6^b^	88.4±2.2^a^	89.5±1.8^a^
Ash (%)	38.2±3.1^b^	41.4±1.9^a^	41.2±6.1^a^
EE (%)	81.3±5.1^b^	87.6±2.2^a^	80.3±7.9^b^
P (%)	44.3±4.1^b^	46.1±6.2^a^	46.2±4.4^a^
Ca (%)	73.3±6.8^b^	78.4±5.1^a^	77.2±1.1^a^

Apparent digestibility values were expressed as percentages (%).

CON: control group; L-PGP: low-dose *Platycodon grandiflorum* polysaccharide group; H-PGP: high-dose *Platycodon grandiflorum* polysaccharide group.

a,b Using p<0.05 as the criterion of significant difference, values with different lowercase letters superscripts mean significant difference.

DM, dry matter; CF, crude fiber; NDF, neutral detergent fiber; CP, crude protein; Ash, crude ash; EE, crude fat; P, phosphorus; Ca, calcium.

## Data Availability

Upon reasonable request, the datasets of this study can be available from the corresponding author.

## SUPPLEMENTARY MATERIAL

**Supplement 1**. Component and nutrition of experimental diet.

**Supplement 2**. Sequences of primers used for qRT-PCR.

**Supplement 3**. Reaction conditions of PCR.

**Supplement 4**. Reaction system of PCR.

## References

[1] Campbell JM Crenshaw JD Polo J The biological stress of early weaned piglets J Anim Sci Biotechnol 2013 4 19 10.1186/2049-1891-4-19 23631414 PMC3651348

[2] Li Y Guo Y Wen Z Jiang X Ma X Han X Weaning stress perturbs gut microbiome and its metabolic profile in piglets Sci Rep 2018 8 18068 10.1038/s41598-018-33649-8 30584255 PMC6305375

[3] Johnson JS Lay DC Jr Evaluating the behavior, growth performance, immune parameters, and intestinal morphology of weaned piglets after simulated transport and heat stress when antibiotics are eliminated from the diet or replaced with L-glutamine J Anim Sci 2017 95 91 102 10.2527/jas.2016.1070 28177383

[4] Wurtz KE Siegford JM Bates RO Ernst CW Steibel JP Estimation of genetic parameters for lesion scores and growth traits in group-housed pigs J Anim Sci 2017 95 4310 7 10.2527/jas2017.1757 29108070

[5] Tuchscherer M Kanitz E Puppe B Tuchscherer A Viergutz T Changes in endocrine and immune responses of neonatal pigs exposed to a psychosocial stressor Res Vet Sci 2009 87 380 8 10.1016/j.rvsc.2009.04.010 19433330

[6] Yin J Wu MM Xiao H Development of an antioxidant system after early weaning in piglets J Anim Sci 2014 92 612 9 10.2527/jas.2013-6986 24352957

[7] Hu CH Xiao K Luan ZS Song J Early weaning increases intestinal permeability, alters expression of cytokine and tight junction proteins, and activates mitogen-activated protein kinases in pigs J Anim Sci 2013 91 1094 101 10.2527/jas.2012-5796 23230104

[8] Xu J Jia Z Xiao S Long C Wang L Effects of enterotoxigenic Escherichia coli challenge on jejunal morphology and microbial community profiles in weaned crossbred piglets Microorganisms 2023 11 2646 10.3390/microorganisms11112646 38004658 PMC10672776

[9] Wen QH Wang LH Zeng XA Niu DB Wang MS Hydroxyl-related differences for three dietary flavonoids as inhibitors of human purine nucleoside phosphorylase Int J Biol Macromol 2018 118 558 98 10.1016/j.ijbiomac.2018.06.045 29894785

[10] Li W Zhang Y Zhao X Fang L Yang T Xie J Optimization of ultrasonic-assisted extraction of Platycodon grandiflorum polysaccharides and evaluation of its structural, antioxidant and hypoglycemic activity Ultrason Sonochem 2023 100 106635 10.1016/j.ultsonch.2023.106635 37839233 PMC10582823

[11] Ke W Flay KJ Huang X Polysaccharides from Platycodon grandiflorus attenuates high-fat diet induced obesity in mice through targeting gut microbiota Biomed Pharmacother 2023 166 115318 10.1016/j.biopha.2023.115318 37572640

[12] Liu Y Sun Q Xu X Platycodon grandiflorus polysaccharides combined with hesperidin exerted the synergistic effect of relieving ulcerative colitis in mice by modulating PI3K/AKT and JAK2/STAT3 signaling pathways Chin J Nat Med 2025 23 848 62 10.1016/S1875-5364(25)60913-7 40653325

[13] Sheng Y Liu G Wang M Lv Z Du P A selenium polysaccharide from Platycodon grandiflorum rescues PC12 cell death caused by H2O2 via inhibiting oxidative stress Int J Biol Macromol 2017 104 393 9 10.1016/j.ijbiomac.2017.06.052 28610929

[14] Song J Liu Q Hao M Zhai X Chen J Effects of neutral polysaccharide from Platycodon grandiflorum on high-fat diet-induced obesity via the regulation of gut microbiota and metabolites Front Endocrinol 2023 14 1078593 10.3389/fendo.2023.1078593 PMC9908743 36777345

[15] National Research Council (NRC) Guide for the care and use of laboratory animals 8th ed National Academies Press 2011 21595115

[16] Prawirodigdo S Gannon NJ Leury BJ Dunshea FR Acid-insoluble ash is a better indigestible marker than chromic oxide to measure apparent total tract digestibility in pigs Anim Nutr 2021 7 64 71 10.1016/j.aninu.2020.07.003 33997333 PMC8110848

[17] Sun G Su W Bao J Dietary full-fat rice bran prevents the risk of heart ferroptosis and imbalance of energy metabolism induced by prolonged cold stimulation Food Funct 2023 14 1530 44 10.1039/d2fo03673h 36655680

[18] Lerminiaux NA Cameron ADS Horizontal transfer of antibiotic resistance genes in clinical environments Can J Microbiol 2019 65 34 44 10.1139/cjm-2018-0275 30248271

[19] Wang J Deng L Chen M Phytogenic feed additives as natural antibiotic alternatives in animal health and production: a review of the literature of the last decade Anim Nutr 2024 17 244 64 10.1016/j.aninu.2024.01.012 38800730 PMC11127233

[20] Wang K Zhang H Han Q Effects of astragalus and ginseng polysaccharides on growth performance, immune function and intestinal barrier in weaned piglets challenged with lipopolysaccharide J Anim Physiol Anim Nutr 2019 104 1096 105 10.1111/jpn.13244 31724241

[21] Wang Q Wang XF Xing T The combined impact of xylo-oligosaccharides and gamma-irradiated Astragalus polysaccharides on growth performance and intestinal mucosal barrier function of broilers Poult Sci 2021 100 100909 10.1016/j.psj.2020.11.075 33518329 PMC7936216

[22] Fei W Yue N Li A Immunomodulatory effects of Lepidium meyenii Walp. Polysaccharides on an immunosuppression model induced by cyclophosphamide J Immunol Res 2022 2022 1210890 10.1155/2022/1210890 35832646 PMC9273403

[23] Zhou R Zhang J Bu W A new role for the spleen: aggravation of the systemic inflammatory response in rats with severe acute pancreatitis Am J Pathol 2019 189 2233 45 10.1016/j.ajpath.2019.07.008 31430464

[24] Howe KL Reardon C Wang A Nazli A McKay DM Transforming growth factor-β regulation of epithelial tight junction proteins enhances barrier function and blocks enterohemorrhagic Escherichia coli O157:H7-induced increased permeability Am J Pathol 2005 167 1587 97 10.1016/s0002-9440(10)61243-6 16314472 PMC1613202

[25] Li JJ Liu ML Lv JN Chen RL Ding K He JQ Polysaccharides from Platycodonis Radix ameliorated respiratory syncytial virus-induced epithelial cell apoptosis and inflammation through activation of miR-181a-mediated Hippo and SIRT1 pathways Int Immunopharmacol 2022 104 108510 10.1016/j.intimp.2021.108510 34999393

[26] Abou-Zeid SM Aljuaydi SH AbuBakr HO Astaxanthin mitigates thiacloprid-induced liver injury and immunotoxicity in male rats Mar Drugs 2021 19 525 10.3390/md19090525 34564187 PMC8467938

[27] Yan H Ma Y Li Y Insulin inhibits inflammation and promotes atherosclerotic plaque stability via PI3K-Akt pathway activation Immunol Lett 2016 170 7 14 10.1016/j.imlet.2015.12.003 26681144

[28] Gresse R Chaucheyras-Durand F Denis S Weaning-associated feed deprivation stress causes microbiota disruptions in a novel mucin-containing in vitro model of the piglet colon (MPigut-IVM) J Anim Sci Biotechnol 2021 12 75 10.1186/s40104-021-00584-0 34078434 PMC8170946

[29] Reiche J Huber O Post-translational modifications of tight junction transmembrane proteins and their direct effect on barrier function Biochim Biophys Acta Biomembr 2020 1862 183330 10.1016/j.bbamem.2020.183330 32376223

[30] Kim SR Park EJ Dusabimana T Platycodon grandiflorus fermented extracts attenuate endotoxin-induced acute liver injury in mice Nutrients 2020 12 2802 10.3390/nu12092802 32933130 PMC7551015

[31] Choi JH Jin SW Han EH Platycodon grandiflorum root-derived saponins attenuate atopic dermatitis-like skin lesions via suppression of NF-κB and STAT1 and activation of Nrf2/ARE-mediated heme oxygenase-1 Phytomedicine 2014 21 1053 61 10.1016/j.phymed.2014.04.011 24854572

[32] Lu N Tan G Tan H Maackiain prevents amyloid-beta–induced cellular injury via priming PKC-Nrf2 pathway Biomed Res Int 2022 2022 4243210 10.1155/2022/4243210 35782063 PMC9242816

[33] Han M Lin J Yang Y Xinshuaining preparation protects H9c2 cells from H2O2-induced oxidative damage through the PI3K/Akt/Nrf-2 signaling pathway Clin Exp Hypertens 2023 45 2131806 10.1080/10641963.2022.2131806 36266998

[34] Zhou L Liu Z Wang Z Astragalus polysaccharides exerts immunomodulatory effects via TLR4-mediated MyD88-dependent signaling pathway in vitro and in vivo Sci Rep 2017 7 44822 10.1038/srep44822 28303957 PMC5355992

[35] Chen M Wei W Wang Z Huo J Wang W Platycodon grandiflorum: from phytochemical diversity to polysaccharides’ prominent bioactivities and edible-medicinal applications Front Nutr 2026 12 1742082 10.3389/fnut.2025.1742082 41601878 PMC12833434

[36] Zhuang L Chen H Zhang S Zhuang J Li Q Feng Z Intestinal microbiota in early life and its implications on childhood health Genom Proteom Bioinform 2019 17 13 25 10.1016/j.gpb.2018.10.002 PMC6522475 30986482

[37] Gaskins HR Collier CT Anderson DB Antibiotics as growth promotants:mode of action Anim Biotechnol 2006 13 29 42 10.1081/abio-120005768 12212942

[38] Wallace TC Guarner F Madsen K Human gut microbiota and its relationship to health and disease Nutr Rev 2011 69 392 403 10.1111/j.1753-4887.2011.00402.x 21729093

[39] Wei Z He Z Wang T Wang X Wang T Long M Lactobacillus salivarius WZ1 inhibits the inflammatory injury of mouse jejunum caused by enterotoxigenic Escherichia coli K88 by regulating the TLR4/NF-κB/MyD88 inflammatory pathway and gut microbiota Microorganisms 2023 11 657 10.3390/microorganisms11030657 36985229 PMC10055675

[40] Wu L Hu Z Luo X Itaconic acid alleviates perfluorooctanoic acid-induced oxidative stress and intestinal damage by regulating the Keap1/Nrf2/HO-1 pathway and reshaping the gut microbiota Int J Mol Sci 2024 25 9826 10.3390/ijms25189826 39337313 PMC11432532

