# Supplementary material for: Dietary supplementation of Platycodon grandiflorum polysaccharides mitigates weaning stress in piglets by modulating intestinal microbiota and improving gut health
Source: Anim Biosci. 2026 Apr 16;39(7):250877. doi: 10.5713/ab.250877 (PMC13353159; doi:10.5713/ab.250877)
Supplement: Supplementary file 1 [file ab-250877-Supplementary-1.pdf]

Supplement 1. Component and nutrition of experimental diet

Ingredients	content (%)
**Basic diet ingredients**	
Corn	73.00
Full-fat soybean meal, puffed	5.00
Soybean meal, de-hulled	15.30
Fish meal	2.00
Soybean oil	1.00
L-Lysine (98%)	0.39
DL-Met (98%)	0.04
L-Thr (98%)	0.12
L-Trp (98%)	0.02
$CaHPO_4$	1.19
Limestone	0.66
NaCl	0.28
Premix[a] (1%)	1.00
**Nutritional level[b]**	
NE（Mcal/kg）	2.50
Crude protein	16.03
Lysine	0.98
Methionine	0.29
Threonine	0.60
Threonine	0.17
Calcium	0.66

Total phosphorus	0.56
Available phosphorus	0.33
Sodium	0.14
Chlorine	0.19

[a] Provided the following per kilogram of diet: 8, 000 IU Vitamin A; 2, 000 IU Vitamin D_3; 30 mg Vitamin E; 2 mg Vitamin K_3; 1.6 mg Vitamin B_1; 5.0 mg Vitamin B_2; 5.0 mg Vitamin B_6; 0.01 mg Vitamin B_{12}; 20 mg pantothenic acid; 0.01 mg folic acid; 0.05 mg biotin; 15 mg niacin; 140 mg Zn; 150 mg Cu; 160 mg Fe; 40 mg Mn; 0.4 mg I; 0.4 mg Se.

[b] Nutritional level were calculated value.
