# Supplementary material for: Dietary supplementation of Platycodon grandiflorum polysaccharides mitigates weaning stress in piglets by modulating intestinal microbiota and improving gut health
Source: Anim Biosci. 2026 Apr 16;39(7):250877. doi: 10.5713/ab.250877 (PMC13353159; doi:10.5713/ab.250877)
Supplement: Supplementary file 2 [file ab-250877-Supplementary-2.pdf]

Supplement 2. Sequences of primers used for qRT-PCR

Gene	Primer sequence （5'to3'）	Product Size （bp）	Genbank No.
KEAP1	F:CGCCTCATCGAGTTCGCTTACAC<br>R:GCACGGACCACACTGTCAATCTG	107	NM_001114671.1
NRF2	F:AGCACAACACATCCCGTCAGAAAC<br>R:GAGCCTGGTTAGGAGCAATGAAGA	134	XM_013984303.2
PKC	F: CCAGGTCCTCAAGAAGATTGCTCA<br>R: GGGTCATCTCCAGAGTGTTCATTC	146	NM_001004027.1
Pi3k	F:CTCTCGGGAGACCATTCCATCATTG<br>R:TTCTTCATTTCCACCTCTGCCCAAG	84	NM_001190422.1
AKT	F:TTCTGGACAAATCTGAGCCCTAAC<br>R:CGACGGATACAGCGGTCAACTTC	121	NM_214127.2
mTOR	F:CGCCTATTTGCCTATCCTGA	83	NM_2143

	CACTC<br>R:GCACGGAAGGGACAGTTCACAG		01.2
myD88	F:CACGCTCGGTGTATGCCTTCTC<br>R:GCAGCTCATTCATCTGGGTGTAGTC	150	NM_214201.1
NF-κB	F: TTCTGGACCGCTTGGGTAAC<br>R: CACCGTTGGGGTGGTTGATA	120	NM_001048232.1
IL1β	F: ACATGCTGAAGGCTCTCCAC<br>R: CAGGGTGGGCGTGTTATCTT	170	NM_214055.1
IL2	F: CGGATGCTTCCAATCTGGGT<br>R: TTCCCTTTTGCCTCAGGGTC	179	NM_001252429.1
IL4	F: TCCAAACTGGCTGTTGCCTTCTTG<br>R: GGGGTGGAAAGGTGTGGAATGC	132	M86923.1
IL10	F: CGGCCCAGTGAAGAGTTTCT<br>R: TGCCTTCGGCATTACGTCTT	151	NM_214041.1

IFN-α	F: CTCGTGAAGGCGGGAATCAT<br>R: GGTGTGCTCCGGTTCAAGAT	113	NM_001005729.1
TNF-γ	F: GTGCTGTTCCCCAACTCTGA<br>R: GCTTTTTGCTGAGGCTGTCC	79	XM_021091967.1
ERK	F: TCTCCTCCCTCCAGCCAATGT<br>R: CTGAAGAGGACCTGGGAGTAGA	156	AY572787.1
JNK	F:GCTCCACGGAGAAGAACTGCTG<br>R:TGAGTGTCTAGGCTCCAGATGTAG	145	NM_214015.2
MAPK	F: ATGACCCCAGTCAATGCCAG<br>R: CAAAGTAGGGCACCTCCCAG	143	NM_0011 9042.2
TLR-4	F: CGGATGCTTCCAATCTGGGT<br>R: TTCCCTTTTGCCTCAGGGTC	135	NM_214127.1
OCLN	F: CCTCCTCCCCTTTCGGACTA<br>R: TCACTTTCCCGTTGGACGAG	70	NM_001163647.2
CLDN	F:	95	NM_0012

1	ATGACCCCAGTCAATGCCAG<br>R:<br>CAAAGTAGGGCACCTCCCAG		44539.1
ZO1	F:<br>TCAAGGTCTGCCGAGACAAC<br>R:<br>ATCACAGTGTGGTAAGCGCA	140	XM_0210<br>98896.1
β-acting	F:<br>ATGGTGAAGGTCGGAGTGAA<br>R:<br>CCGTGGGTGGAATCATACTG	155	NM_0012<br>06359.1

Supplement 3. Reaction conditions of PCR

PCR reaction temperature	PCR reaction time
98°C	30 s
98°C	10 s
54°C	30 s
72°C	45 s
72°C	10 min
4°C	15 min

Supplement 4. Reaction system of PCR

PCR reaction component	PCR reaction volume
Pusion Hot start flex 2X Master Mix	12.5 μL
