# Supplementary material for: Dietary supplementation of Platycodon grandiflorum polysaccharides mitigates weaning stress in piglets by modulating intestinal microbiota and improving gut health
Source: Anim Biosci. 2026 Apr 16;39(7):250877. doi: 10.5713/ab.250877 (PMC13353159; doi:10.5713/ab.250877)
Supplement: Supplementary file 3 [file ab-250877-Supplementary-3.pdf]

1	ATGACCCCAGTCAATGCCAG<br>R:<br>CAAAGTAGGGCACCTCCCAG		44539.1
ZO1	F:<br>TCAAGGTCTGCCGAGACAAC<br>R:<br>ATCACAGTGTGGTAAGCGCA	140	XM_0210<br>98896.1
β-<br>acting	F:<br>ATGGTGAAGGTCGGAGTGAA<br>R:<br>CCGTGGGTGGAATCATACTG	155	NM_0012<br>06359.1

Supplement 3. Reaction conditions of PCR

PCR reaction temperature	PCR reaction time
98°C	30 s
98°C	10 s
54°C	30 s
72°C	45 s
72°C	10 min
4°C	15 min

Supplement 4. Reaction system of PCR

PCR reaction component	PCR reaction volume
Pusion Hot start flex 2X Master Mix	12.5 μL
