# Supplementary material for: Dietary supplementation of Platycodon grandiflorum polysaccharides mitigates weaning stress in piglets by modulating intestinal microbiota and improving gut health
Source: Anim Biosci. 2026 Apr 16;39(7):250877. doi: 10.5713/ab.250877 (PMC13353159; doi:10.5713/ab.250877)
Supplement: Supplementary file 4 [file ab-250877-Supplementary-4.pdf]

1	ATGACCCCAGTCAATGCCAG<br>R:<br>CAAAGTAGGGCACCTCCCAG		44539.1
ZO1	F:<br>TCAAGGTCTGCCGAGACAAC<br>R:<br>ATCACAGTGTGGTAAGCGCA	140	XM_0210<br>98896.1
β-<br>acting	F:<br>ATGGTGAAGGTCGGAGTGAA<br>R:<br>CCGTGGGTGGAATCATACTG	155	NM_0012<br>06359.1

Supplement 3. Reaction conditions of PCR

PCR reaction temperature	PCR reaction time
98°C	30 s
98°C	10 s
54°C	30 s
72°C	45 s
72°C	10 min
4°C	15 min

Supplement 4. Reaction system of PCR

PCR reaction component	PCR reaction volume
Pusion Hot start flex 2X Master Mix	12.5 μL

Forward Primer	2.5 μL
Reverse Primer	2.5 μL
Template DNA	50 ng
Add H2O to make up to	25 μL
